# Cardiovascular risk factors mediating the protective effect of education on cervical spondylosis risk

**DOI:** 10.1038/s41598-023-28153-7

**Published:** 2023-01-17

**Authors:** Yang Sun, Manqiu Jin, Tiecheng Yu, Jiting Zhang

**Affiliations:** 1 Department of Orthopedics, The First Hospital of Jilin University, Jilin Changchun, China; 2 Department of Plastic Surgery, The First Hospital of Jilin University, Jilin Changchun, China

**Keywords:** Health care, Risk factors, Cardiovascular diseases, Musculoskeletal system

## Abstract

The causal association between education and cervical spondylosis may be mediated partly through risk factors of cardiovascular disease. The identification of the protective effect of education and the evaluation of risk factors will help to optimize disease prevention at both clinical and public health levels. In this study, we applied several different Mendelian randomization (MR) methods to identify which cardiovascular factors underlie the clustering of cervical spondylosis with cardiovascular disease, and the degree to which these mediate an effect of education. Univariable MR analyses provided evidence supporting a protective effect of genetically predicted education on cervical spondylosis risk, and MVMR further identified the direct effect of education level. Our results also provided evidence supporting the detrimental effects of BMI and smoking on cervical spondylosis risk, with evidence that the effect of education is mediated through BMI and smoking. The proportions of the effect of education mediated through BMI and smoking were 12% and 3%, respectively. These findings highlight education, obesity, and smoking as common mechanisms underlying the clustering of cervical spondylosis with risk factors of cardiovascular disease, which might represent clinical and public health targets for reducing multi-morbidity and the burden of these common conditions.

## Introduction

Cervical spondylosis is a chronic, developing deterioration of osseocartilaginous components of the cervical spine that is most commonly associated with aging, and disease-modifying agents are not currently available^1^. According to population-based research, by the age of 50, approximately 80–90% of people have disk degeneration^2,3^. A survey of the global burden of low back and neck pain reported that in 2015, more than a third of a billion people worldwide suffered from mechanical neck pain for at least 3 months^4^, indicating the global health implications of degenerative cervical spondylosis^5^. During the last years, close relationship was observed between cervical spondylosis and cardiovascular diseases such as acute coronary syndrome, arrhythmia, and hypertension, which had attracted more and more close attention^6–8^. An elevated rate of cardiovascular disease is observed in cervical spondylosis^7,9,10^. A total of 744 acute coronary syndrome events were identified among the 27,948 patients with cervical spondylosis^7^. The overall incidence of acute coronary syndrome was 4.27 per 1000 person-years in the cervical spondylosis cohort and 3.90 per 1000 person-years in the non-CS cohort^7^. It is also found that lower educational levels are linked to higher rates of cardiovascular disease^11,12^. There is, however, a lack of understanding of the underlying mechanisms. The causal association between education and cervical spondylosis may be mediated partly through risk factors of cardiovascular disease^11,12^. The identification of the protective effect of education and the evaluation of risk factors will help to optimize disease prevention at both clinical and public health levels.

Mendelian randomization (MR)^13^ has proven to be a reliable method for overcoming the limitations of observational studies and assessing causality. As genes are randomly assigned at conception, their association with the outcome is less influenced by external confounders. Given that certain exposures are correlated and pleiotropic, we further leveraged multivariable MR (MVMR) methods developed in recent years^14^ to adjust for potential pleiotropy. Recently, MR methods have been used to investigate mediating pathways^15,16^. This method retains the advantages of using genetic variants for causal inference, such as reducing bias caused by confounders, while allowing for estimation of the different effects required for mediation analysis.

Our study sought to evaluate the effects of education and cardiovascular risk factors on the risk of cervical spondylosis by using the MR framework. For cardiovascular risk factors that showed a causal effect on cervical spondylosis risk, we further performed the MR mediation analyses to estimate the extent to which these factors might be mediating the effects of education.

## Methods

### Overall study design

**Figure 1:**
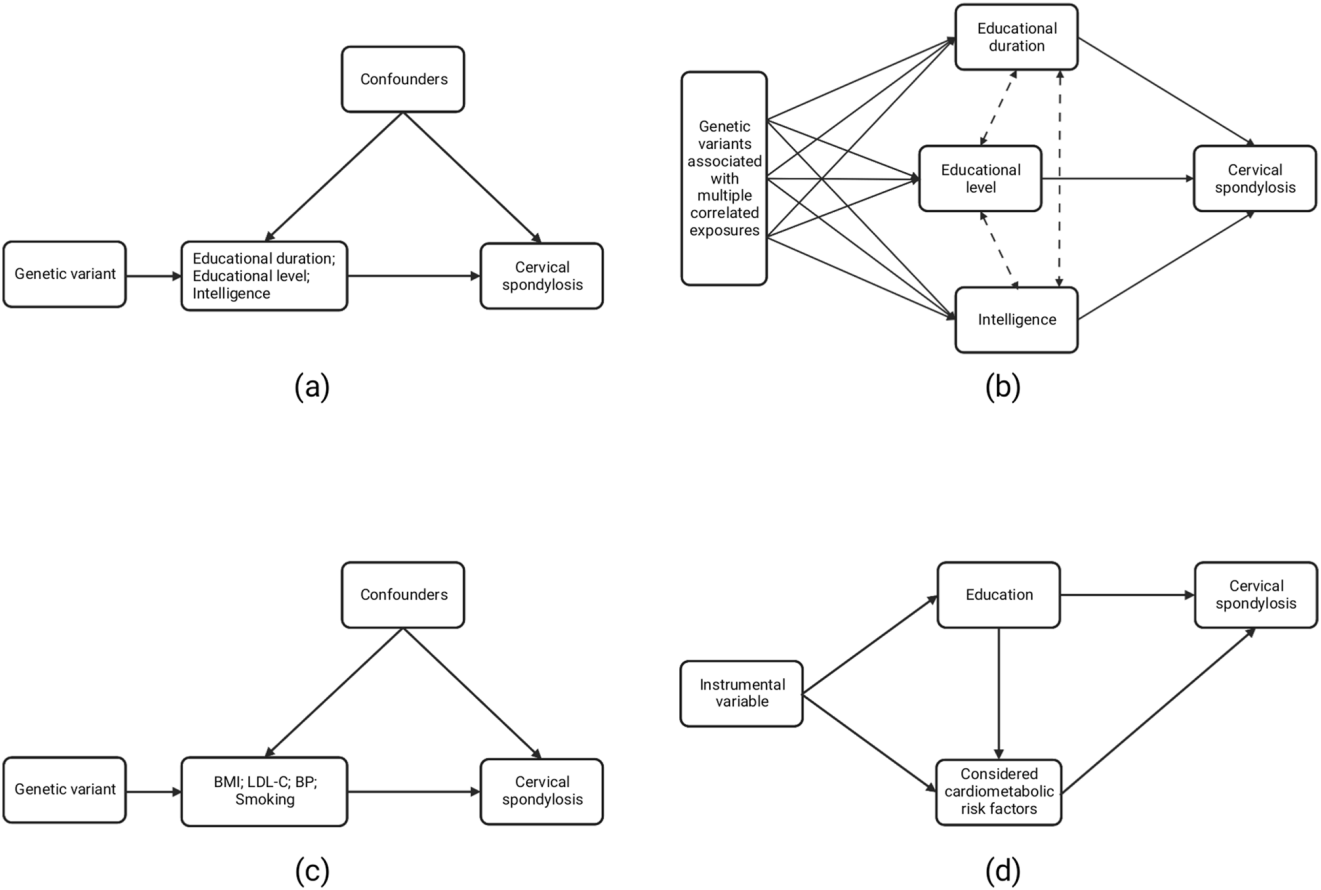
Schematic representation of overall study design. (**a**) Schematic representation of univariable Mendelian randomization analysis for education. (**b**) Schematic representation of multivariable Mendelian randomization analysis for education. (**c**) Schematic representation of univariable Mendelian randomization analysis for risk factors of cardiovascular disease. (**d**) Schematic representation of mediation analysis of the mediation effect of considered cardiovascular risk factors on education–cervical spondylosis risk.

In this study, we have extracted instrumental variables (IVs) for education (including educational duration, educational level, and intelligence) from publicly available summary statistics, and assessed the causal effects of education on the risk of cervical spondylosis by applying univariable and multivariable MR analyses. Additionally, we also evaluated the effects of common risk factors of cardiovascular disease, including blood pressure (BP), low-density lipoprotein cholesterol (LDL-C), body mass index (BMI), and smoking on cervical spondylosis. For cardiovascular risk factors for which there was MR evidence of an unfavorable effect of their genetically predicted levels on cervical spondylosis risk, we further conducted a mediation analysis to estimate the proportion of education’s effect on cervical spondylosis mediated through considered cardiovascular risk factors. The schematic was shown in Fig. 1. Since no primary data were used in this study, ethical approval was not required.

### Data source

The characteristics of each contributing study are presented in Table 1. The summary statistics data on cervical spondylosis were retrieved from the FinnGen biobank, which included 171,956 Europeans totaling around 16,380,237 single nucleotide polymorphisms (SNPs) with acceptable imputation quality. The trait of this study was labelled "Cervical disc disorders" and the category of variable was binary. Genetic variants associated with education were obtained from the UK Biobank. We identified 237 SNPs associated with educational level, 22 SNPs with educational duration, and 49 SNPs with intelligence at a genome-wide significance level. The educational level was defined as "Qualifications: College or University degree" and its variable category was binary; the educational duration was defined as "Age completed full time education" and its variable category was categorical ordered; and the trait of intelligence was labelled "Fluid intelligence score", whose category was categorical ordered. Summary statistics for BMI, LDL-C, and BP were acquired from the UK Biobank, which were all measured as continuous. Genetic variants for smoking were retrieved from a genome-wide association studies (GWAS) of 607,291 Europeans in the GWAS & Sequencing Consortium of Alcohol and Nicotine use (GSCAN). There are multiple stages of tobacco use (initiation, cessation, and heaviness). In this study, the smoking was defined as "smoking initiation". The smoking initiation phenotypes included age of initiation of regular smoking and a binary phenotype indicating whether an individual had ever smoked regularly.

**Table 1:** Details of the instruments used for exposures.

Exposure	Consortium	Sample size	No. SNPs	I^2^	F-statistics	Pleiotropy		Heterogeneity		Population
						Intercept	P value	Q	P value	
Educational duration	UK Biobank	307,897	22	8.927E−04	14.049	1.229E−02	0.518	30.675	0.060	European
Educational level	UK Biobank	458,079	237	7.216E−03	14.042	− 7.357E−03	0.261	261.957	0.109	European
Intelligence	UK Biobank	149,051	49	4.824E−03	14.741	2.299E−03	0.869	57.141	0.148	European
BMI	UK Biobank	461,460	660	2.780E−02	19.967	1.914E−04	0.939	755.199	0.005	European
LDL-C	UK Biobank	440,546	250	1.993E−02	35.821	− 2.677E−04	0.914	343.492	0.003	European
BP	UK Biobank	336,683	155	9.052E−03	19.833	− 5.716E−03	0.357	195.820	0.002	European
Smoking	GSCAN	607,291	155	5.018E−03	19.756	1.737E−02	0.127	190.689	0.018	European

*No.SNPs* number of single nucleotide polymorphism, *MRC-IEU* medical research council-integrative epidemiology unit, *BMI* body mass index, *LDL-C* low density lipoprotein-cholesterol, *BP* blood pressure, *GSCAN* GWAS and sequencing consortium of alcohol and nicotine use.

### Statistical analysis

TwoSampleMR and MendelianRandomization R packages were used for all analyses. All genetic variants reaching genome-wide significance (p < 5 × 10e-8) and being independent (5000 kb pairs apart and R^2^ ≤ 0.01) were selected as instruments for the MR analysis. The exposure and outcome GWAS provided SNP effects and corresponding standard errors^17^. We filtered out palindromic SNPs with intermediate allele frequencies after harmonizing exposure and outcome data^18^. We used the PhenoScanner V2 database to detect potential pleiotropy among the SNPs included in this study^19^. Genetic variants that were found to be related to potential confounding factors were excluded from the analyses. And F statistic was calculated to assess the strength of the selected SNPs.

For univariable MR analysis, the inverse variance–weighted (IVW) method was identified as the primary MR analysis. Weighted median and MR-Egger-based regression methods were incorporated to ensure the conclusions were reliable since the IVW method provides consistent estimates only when all genetic variants are valid IVs^13,17,20^. When 100% genetic variants are invalid IVs, the MR-Egger regression provides reliable estimates; in contrast, the weighted median requires that 50% of the weight come from valid IVs. In terms of efficiency, however, weighted median estimates are typically almost as accurate as IVW estimates; both are significantly more accurate than MR-Egger estimates, with MR-Egger regression estimates being especially inaccurate when all IVs are associated with exposure to similar magnitudes^21^. To assess potential IV pleiotropy, we also conducted the MR pleiotropy residual sum and outlier (MR-PRESSO) test^22^, MR-Egger intercept test^23^, and Cochran Q heterogeneity test^24^. The "leave-one-out" sensitivity analyses were also performed to detect potentially influential SNPs^25^.

Multivariable MR (MVMR) is a novel extension to MR that incorporates genetic variants associated with multiple, potentially correlated exposures to calculate the effect of each exposure on a single outcome^26^. For this approach, the genetic variants do not have to be exclusively linked to a single exposure, but with a set of measured exposures, although it still needs to meet equivalent instrumental-variable assumptions^14^. The method gives a direct causal estimation for each exposure, taking into account the association between that exposure and the IVs with the other exposures in the analysis.

MVMR permits for equivalent analysis to mediation within the MR framework and therefore could also be applied to quantify mediation effects^26^. To investigate the potential mechanisms for the effect of education on cervical spondylosis, we estimated the causal associations between cardiovascular risk factors and cervical spondylosis. Summary level statistics of the potential mediators were extracted from GWAS based on subjects not overlapping with the outcome. Table 1 provides detailed information on their data sources. With MVMR, the direct effects of education on cervical spondylosis were estimated after adjusting for risk factors^27^. Then, we calculated the indirect effect and estimated the proportion of education’s effect on cervical spondylosis mediated through considered cardiovascular risk factors.

## Results

All IVs analyzed in the univariable and MVMR analyses are presented in Supplementary Tables S1–S13 and visualized in Supplementary Figs. S1–S7. The intercept of the MR-Egger regression indicated that there was no directional pleiotropy among the SNPs associated with exposures (Table 1). The F-statistics of IVs ranged between 14.042 and 35.821, all > 10, indicating no evidence of weak instrument bias (Table 1).

**Figure 2:**
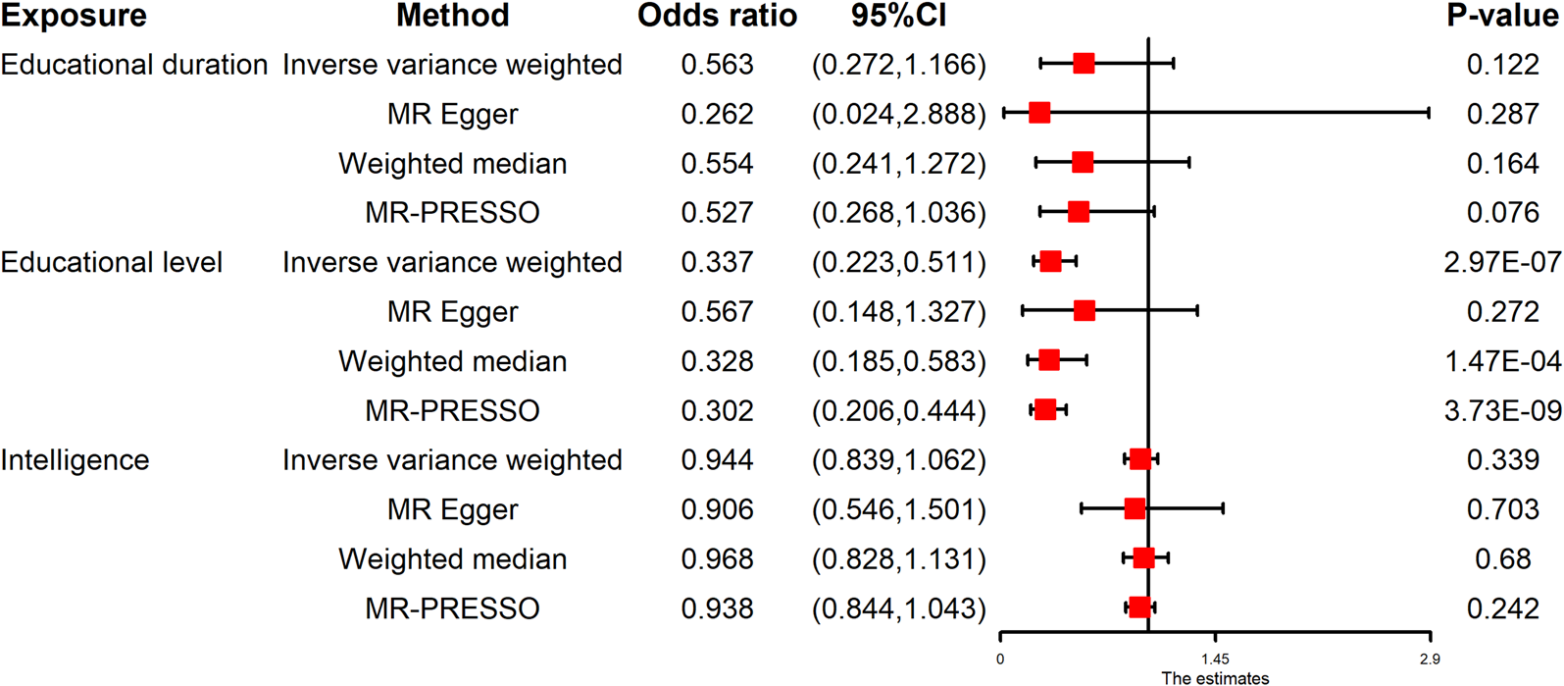
Forest plot for the univariable MR analyses of the causal effect of education on cervical spondylosis. The education-related traits include educational duration, educational level, and intelligence. *MR* Mendelian randomization, *CI* confidence interval.

**Figure 3:**
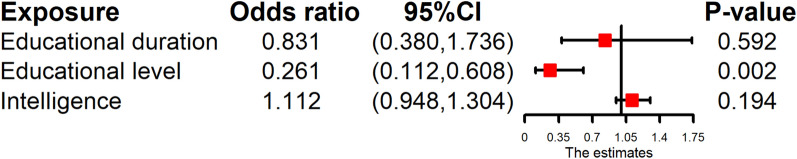
Forest plot for the MVMR analyses of the causal effect of education on cervical spondylosis. The education-related traits include educational duration, educational level, and intelligence. *MVMR* multivariable Mendelian randomization, *CI* confidence interval.

The univariable MR provided evidence of a protective effect of education on cervical spondylosis risk in the primary IVW analyses (educational duration: OR 0.563, 95% CI 0.272–1.166, P = 0.122; educational level: OR 0.337, 95% CI 0.223–0.511, P = 2.97E−07; intelligence: OR 0.944, 95% CI 0.839–1.062, P = 0.339), with consistent findings in sensitivity analyses (Fig. 2). The “leave-one-out analysis” plots were presented in Supplementary Figs. S8–S10. After adjusting for the exposures by MVMR, we found the exposure mainly associated with cervical spondylosis was educational level (educational level: OR 0.261, 95% CI 0.112–0.608, P = 1.85E−03) (Fig. 3).

**Figure 4:**
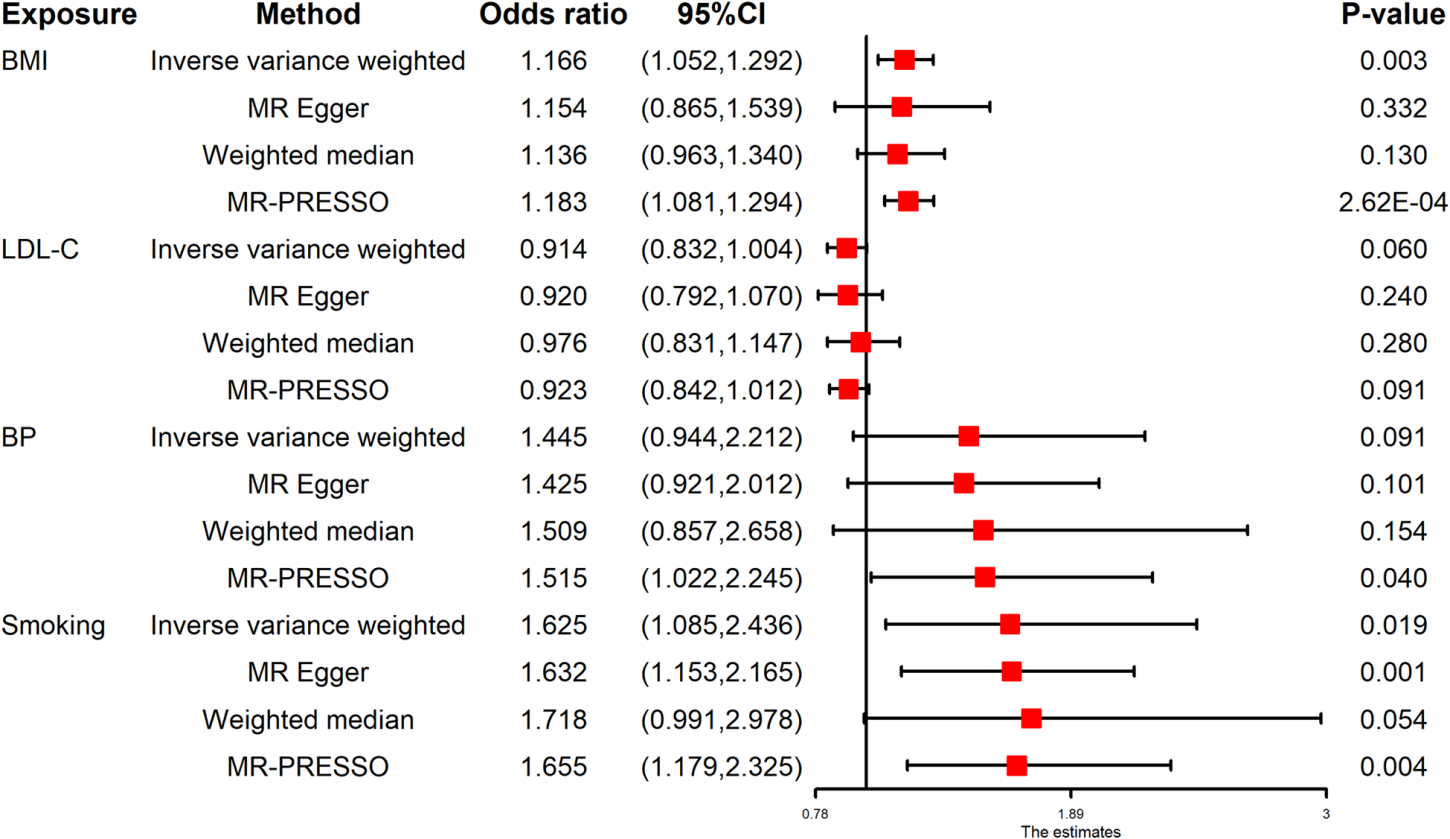
Forest plot for the univariable MR analyses of the causal effect of cardiovascular risk factors on cervical spondylosis. *BMI* body mass index, *LDL-C* low-density lipoprotein cholesterol, *BP* blood pressure, *MR* Mendelian randomization, *CI* confidence interval.

In the univariable MR, there was evidence of a detrimental effect of BMI and smoking on cervical spondylosis risk in the primary IVW analyses (BMI: OR 1.166, 95% CI 1.052–1.292, P = 0.003; smoking: OR 1.625, 95% CI 1.085–2.436, P = 0.019) (Fig. 4). The “leave-one-out analysis” plots were presented in Supplementary Figs. S11 and S14. However, MR estimates gave little support to a possible causal effect of LDL-C and BP on cervical spondylosis (LDL-C: OR 0.914, 95% CI 0.832–1.004, P = 0.060; BP: OR 1.445, 95% CI 0.944–2.212, P = 0.091), with consistent findings in sensitivity analyses (Fig. 4). The “leave-one-out analysis” plots were presented in Supplementary Figs. S12 and S13.

**Figure 5:**
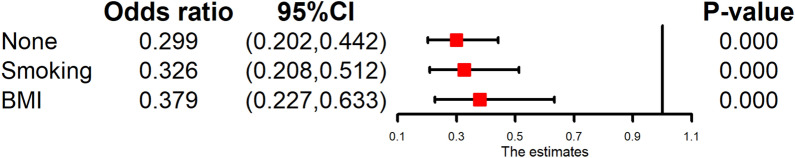
Forest plot for the mediation analysis of the mediation effect of smoking and BMI on education–cervical spondylosis risk. *CI* confidence interval.

Given the identified effects of higher genetically predicted BMI and higher genetically predicted smoking on increasing cervical spondylosis risk, MVMR mediation analyses were conducted to evaluate the extent to which these factors were mediating the effect of genetically predicted education (educational level) on cervical spondylosis risk. The protective effect of genetically predicted education (educational level) on cervical spondylosis risk attenuated from OR of 0.299 (95% CI 0.202–0.442) in IVW univariable analysis to OR of 0.326 (95% CI 0.208–0.512) after adjusting for smoking in MVMR analysis, and to OR of 0.379 (95% CI 0.227–0.633) after adjusting for BMI in MVMR analysis (Fig. 5). The proportion of the effect of genetically predicted education mediated through BMI and smoking was estimated as 12% (95% CI 2–22%) and 3% (95% CI 1–7%), respectively.

## Discussion

This study utilizes large-scale GWAS statistics to evaluate the causal effects of genetically predicted education and cardiovascular risk factors on cervical spondylosis in an MR framework and demonstrates the protective effects of education and the adverse effects of smoking and BMI. Our results provide new insight into the causal mechanisms underlying cervical spondylosis, its clustering with the risk factors of cardiovascular disease, and disparities related to educational attainment.

The findings are consistent with earlier observational studies that found smoking and BMI had a detrimental impact on the risk of cervical spondylosis^28,29^. However, our present study takes a further step to uncover a causality between genetically predicted education and risk for cervical spondylosis, as well as to quantify how much genetically predicted BMI and smoking mediate this association. Our results show relevance in both clinical and public health aspects. In addition to cardiovascular disease, smoking and obesity have widespread effects on human health. Obesity is a major risk factor for diabetes, while smoking is associated with chronic lung disease and many cancers^30^. Targeting these risk factors not only interrupts the course of many common diseases but also reduces the burden of multi-morbidity on individuals and healthcare systems^30^. The identification of obesity and smoking as downstream mediators of education confirms that policies to improve educational levels should proceed^12,31^. It is known that educational attainment is heritable, and by applying IVs closely related to this trait, we could investigate its associations with cervical spondylosis risk. Earlier studies suggest that education experience is more likely to influence health outcomes than cognitive ability in a related context^32^. Our study found that education protects against cervical spondylosis risk by reducing smoking and BMI. The estimates, however, contained some uncertainty. Comparatively, it has been estimated that blood pressure, obesity, and smoking together mediate about half of the protective effect of education on cardiovascular disease^11^.

There are limitations to our study. Firstly, the study population only included individuals of European lineage. More studies should be conducted to verify the applicability of these results to other ethnicities. Secondly, the potential horizontal pleiotropy cannot be controlled. Thirdly, due to limited resources, the most recent individual-level statistics are not available for our study.

In summary, our work uses summary statistics from publicly available GWASs in the MR framework to provide evidence supporting a protective effect of education and unfavorable effects of smoking and BMI on cervical spondylosis risk, with findings that the effect of education is partly mediated through smoking and BMI. Our results indicate education, obesity, and smoking as common mechanisms underlying the clustering of cervical spondylosis with cardiovascular disease risk factors, which may represent clinical and public health targets for reducing multi-morbidity and the burden of these common diseases.

## Supplementary Information

Supplementary Information 1.

Supplementary Information 2.

## Data Availability

The data that support the findings presented in this study are available from the corresponding authors upon reasonable request.

## References

[1] Theodore N (2020). Degenerative cervical spondylosis. N. Engl. J. Med..

[2] Brinjikji W (2015). Systematic literature review of imaging features of spinal degeneration in asymptomatic populations. AJNR Am. J. Neuroradiol..

[3] Teraguchi M (2014). Prevalence and distribution of intervertebral disc degeneration over the entire spine in a population-based cohort: The Wakayama Spine Study. Osteoarth. Cartil..

[4] Hurwitz EL, Randhawa K, Yu H, Côté P, Haldeman S (2018). The Global Spine Care Initiative: A summary of the global burden of low back and neck pain studies. Eur. Spine J..

[5] Global, regional, and national incidence, prevalence, and years lived with disability for 301 acute and chronic diseases and injuries in 188 countries, 1990–2013: A systematic analysis for the Global Burden of Disease Study 2013. *Lancet (London, England)* **386**, 743–800. 10.1016/s0140-6736(15)60692-4 (2015). 10.1016/S0140-6736(15)60692-4 PMC4561509 26063472

[6] He ZB (2017). Atlantoaxial misalignment causes high blood pressure in rats: A novel hypertension model. Biomed. Res. Int..

[7] Lin SY (2018). Risk of acute coronary syndrome in patients with cervical spondylosis. Atherosclerosis.

[8] Lin, S. Y. *et al.* Association of Arrhythmia in Patients with Cervical Spondylosis: A nationwide population-based cohort study. *J. Clin. Med.* **7**. doi:10.3390/jcm7090236 (2018). 10.3390/jcm7090236 PMC6162845 30142924

[9] Fineberg SJ, Oglesby M, Patel AA, Singh K (2013). Incidence and mortality of perioperative cardiac events in cervical spine surgery. Spine.

[10] Oglesby M, Fineberg SJ, Patel AA, Pelton MA, Singh K (2013). The incidence and mortality of thromboembolic events in cervical spine surgery. Spine.

[11] Carter AR (1855). Understanding the consequences of education inequality on cardiovascular disease: Mendelian randomisation study. BMJ (Clin. Res. ed.).

[12] Tillmann, T. *et al.* Education and coronary heart disease: Mendelian randomisation study. *BMJ (Clinical research ed.)* **358**, j3542. 10.1136/bmj.j3542 (2017). 10.1136/bmj.j3542 PMC5594424 28855160

[13] Smith GD, Ebrahim S (2003). 'Mendelian randomization': Can genetic epidemiology contribute to understanding environmental determinants of disease?. Int. J. Epidemiol..

[14] Sanderson, E., Davey Smith, G., Windmeijer, F. & Bowden, J. An examination of multivariable Mendelian randomization in the single-sample and two-sample summary data settings. *Int. J. Epidemiol.* **48**, 713–727. 10.1093/ije/dyy262 (2019). 10.1093/ije/dyy262 PMC6734942 30535378

[15] Burgess S (2017). Dissecting causal pathways using Mendelian randomization with summarized genetic data: Application to age at menarche and risk of breast cancer. Genetics.

[16] Carter AR (2021). Mendelian randomisation for mediation analysis: Current methods and challenges for implementation. Eur. J. Epidemiol..

[17] Hemani, G. *et al.* The MR-Base platform supports systematic causal inference across the human phenome. *eLife* **7**. 10.7554/eLife.34408 (2018). 10.7554/eLife.34408 PMC5976434 29846171

[18] Burgess S, Davies NM, Thompson SG (2016). Bias due to participant overlap in two-sample Mendelian randomization. Genet. Epidemiol..

[19] Staley JR (2016). PhenoScanner: A database of human genotype-phenotype associations. Bioinformatics (Oxford, England).

[20] Bowden J, Davey Smith G, Burgess S (2015). Mendelian randomization with invalid instruments: Effect estimation and bias detection through Egger regression. Int. J. Epidemiol..

[21] Bowden, J., Davey Smith, G., Haycock, P. C. & Burgess, S. Consistent Estimation in Mendelian Randomization with Some Invalid Instruments Using a Weighted Median Estimator. *Genet. Epidemiol.* **40**, 304–314. 10.1002/gepi.21965 (2016). 10.1002/gepi.21965 PMC4849733 27061298

[22] Verbanck M, Chen CY, Neale B, Do R (2018). Detection of widespread horizontal pleiotropy in causal relationships inferred from Mendelian randomization between complex traits and diseases. Nat. Genet..

[23] Bowden J (2017). A framework for the investigation of pleiotropy in two-sample summary data Mendelian randomization. Stat. Med..

[24] Bowden J (2019). Improving the accuracy of two-sample summary-data Mendelian randomization: moving beyond the NOME assumption. Int. J. Epidemiol..

[25] Hemani, G., Bowden, J. & Davey Smith, G. Evaluating the potential role of pleiotropy in Mendelian randomization studies. *Hum. Mol. Genet.* **27**, R195–R208. 10.1093/hmg/ddy163 (2018). 10.1093/hmg/ddy163 PMC6061876 29771313

[26] Sanderson, E. Multivariable Mendelian Randomization and Mediation. *Cold Spring Harbor Perspect. Med.* **11**. 10.1101/cshperspect.a038984 (2021). 10.1101/cshperspect.a038984 PMC7849347 32341063

[27] Sanderson E, Smith GD, Windmeijer F, Bowden J (2020). Corrigendum to: An examination of multivariable Mendelian randomization in the single-sample and two-sample summary data settings. Int. J. Epidemiol..

[28] Chen Z, Li X, Pan F, Wu D, Li H (2018). A retrospective study: Does cigarette smoking induce cervical disc degeneration?. Int. J. Surg. (London, England).

[29] He LC (2014). Prevalence and risk factors of lumbar spondylolisthesis in elderly Chinese men and women. Eur. Radiol..

[30] Stewart, S. T., Cutler, D. M. & Rosen, A. B. Forecasting the effects of obesity and smoking on U.S. life expectancy. *N. Engl. J. Med.* **361**, 2252–2260. 10.1056/NEJMsa0900459 (2009). 10.1056/NEJMsa0900459 PMC4394736 19955525

[31] Di Chiara, T. *et al.* Association between low education and higher global cardiovascular risk. *J. Clin. Hypertension (Greenwich, Conn.)* **17**, 332–337. 10.1111/jch.12506 (2015). 10.1111/jch.12506 PMC8031686 25703272

[32] Gill D, Efstathiadou A, Cawood K, Tzoulaki I, Dehghan A (2019). Education protects against coronary heart disease and stroke independently of cognitive function: Evidence from Mendelian randomization. Int. J. Epidemiol..

