# Supplementary material for: Cardiovascular risk factors mediating the protective effect of education on cervical spondylosis risk
Source: Sci Rep. 2023 Jan 17;13:936. doi: 10.1038/s41598-023-28153-7 (PMC9845322; doi:10.1038/s41598-023-28153-7)
Supplement: Supplementary file 1 — Supplementary Information 1. [file 41598_2023_28153_MOESM1_ESM.pdf]

# Supplementary Figures

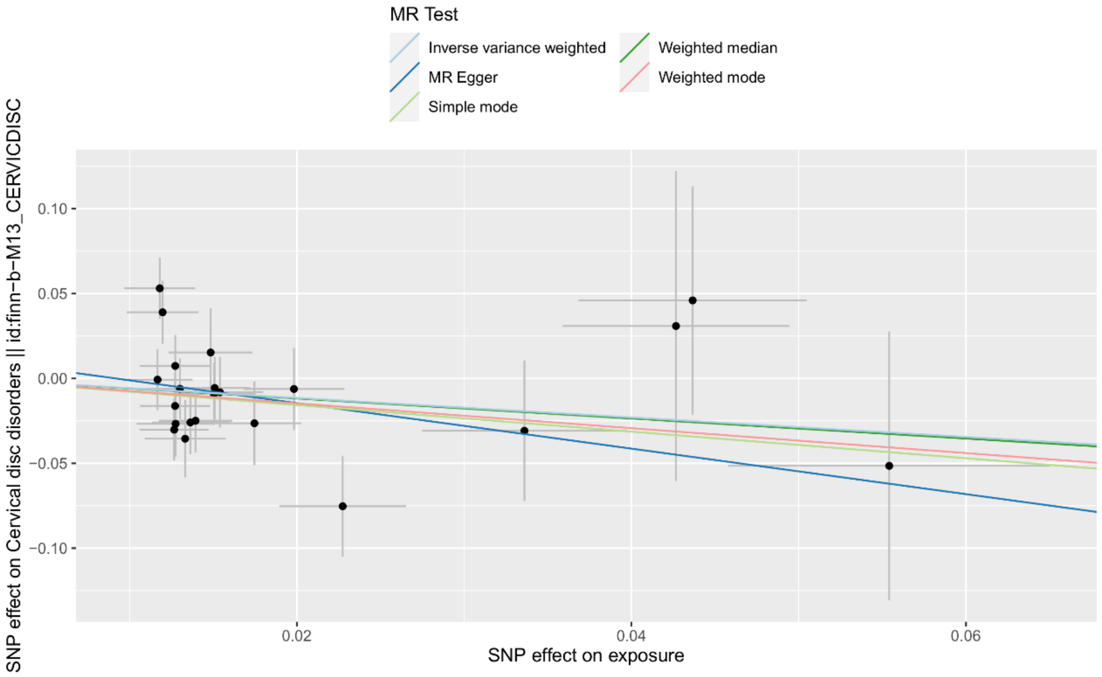


**Supplementary Figure 1.** A scatterplot of variant-educational duration (x-axis) and variant-cervical spondylosis risk (y-axis) association estimates for educational duration instruments in univariable MR analysis. The slope of each line corresponds to the effect size obtained by the corresponding MR method.

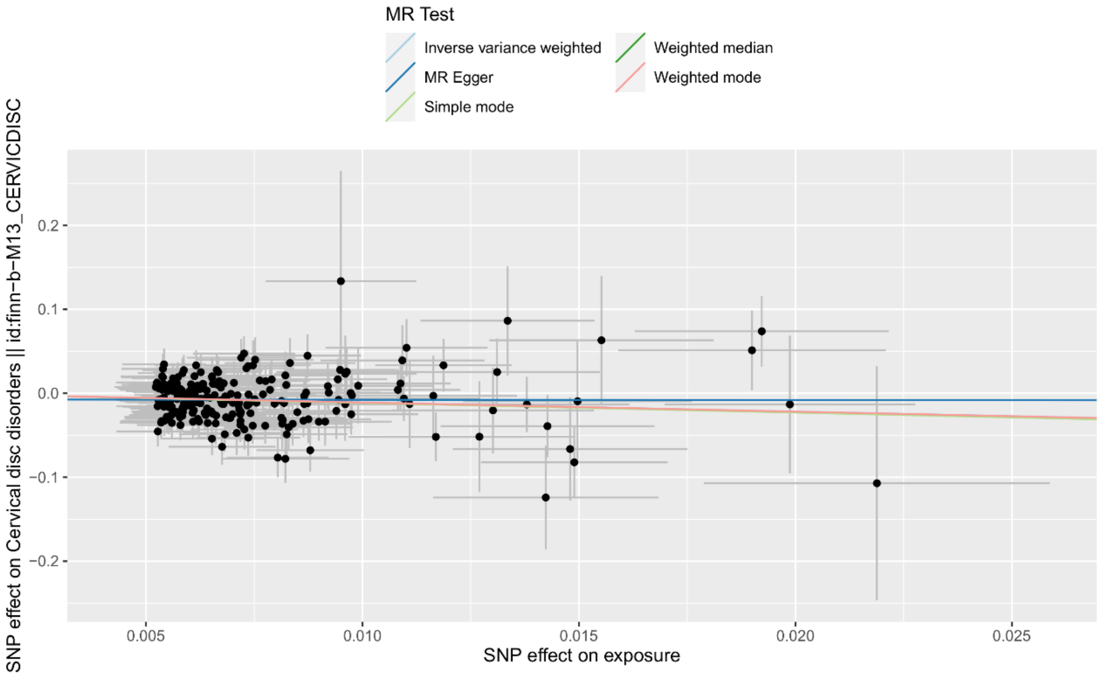


**Supplementary Figure 2.** A scatterplot of variant-educational level (x-axis) and variant-cervical spondylosis risk (y-axis) association estimates for educational level instruments in univariable MR analysis. The slope of each line corresponds to the effect size obtained by the corresponding MR method.

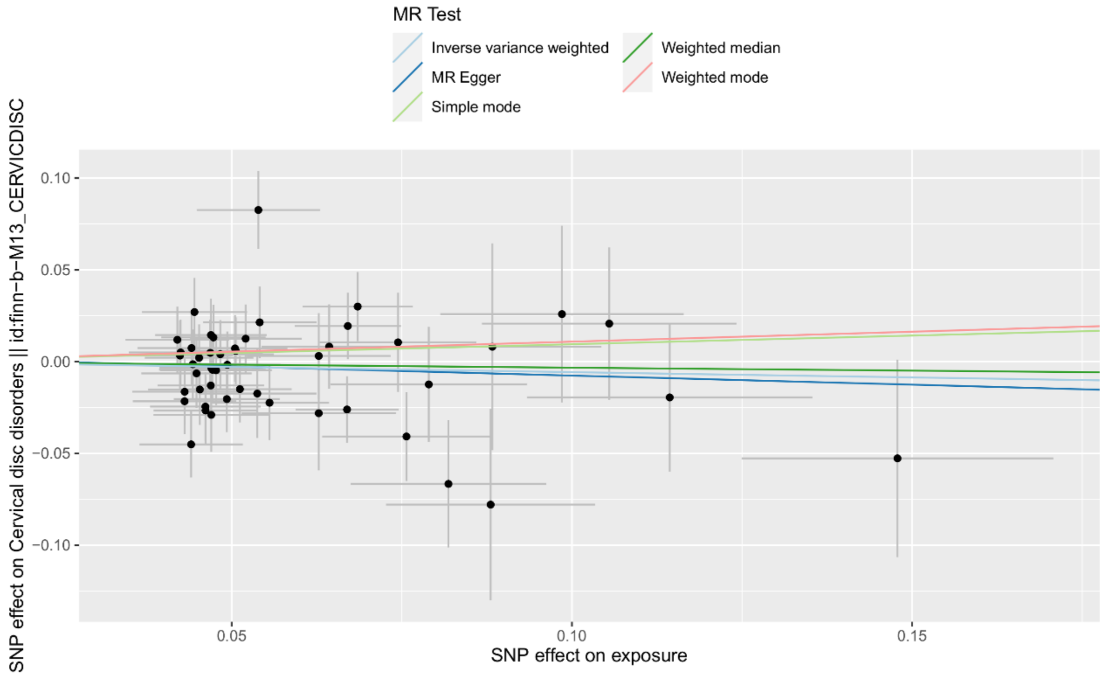


**Supplementary Figure 3.** A scatterplot of variant-intelligence (x-axis) and variant-cervical spondylosis risk (y-axis) association estimates for intelligence instruments in univariable MR analysis. The slope of each line corresponds to the effect size obtained by the corresponding MR method.

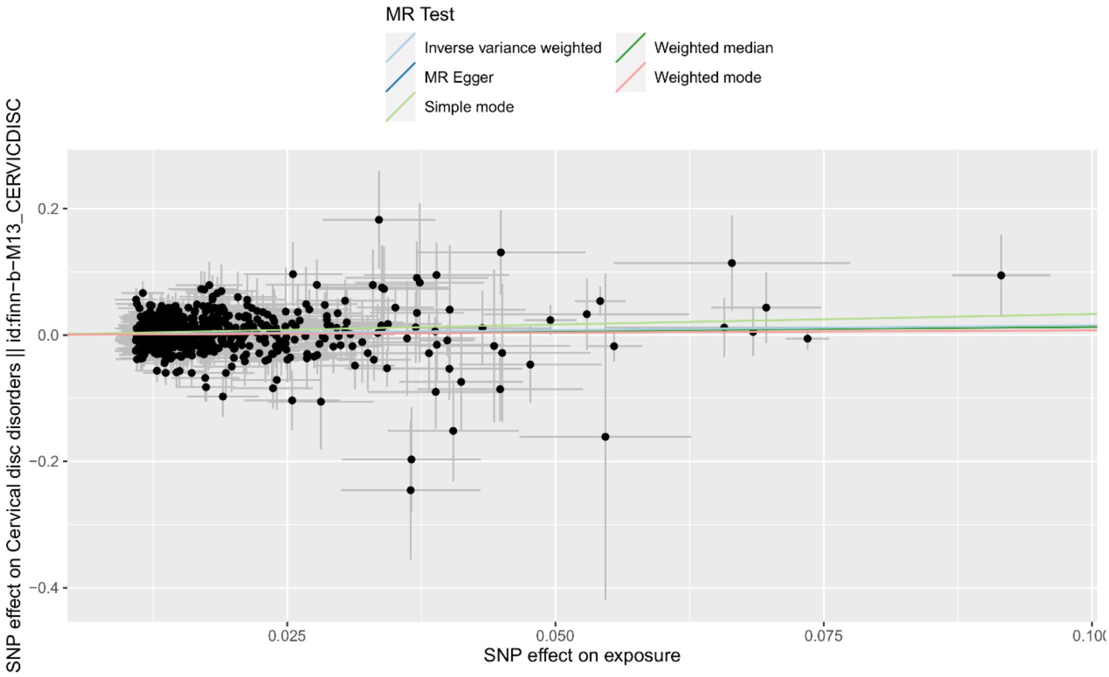


**Supplementary Figure 4.** A scatterplot of variant-BMI (x-axis) and variant-cervical spondylosis risk (y-axis) association estimates for BMI instruments in univariable MR analysis. The slope of each line corresponds to the effect size obtained by the corresponding MR method.

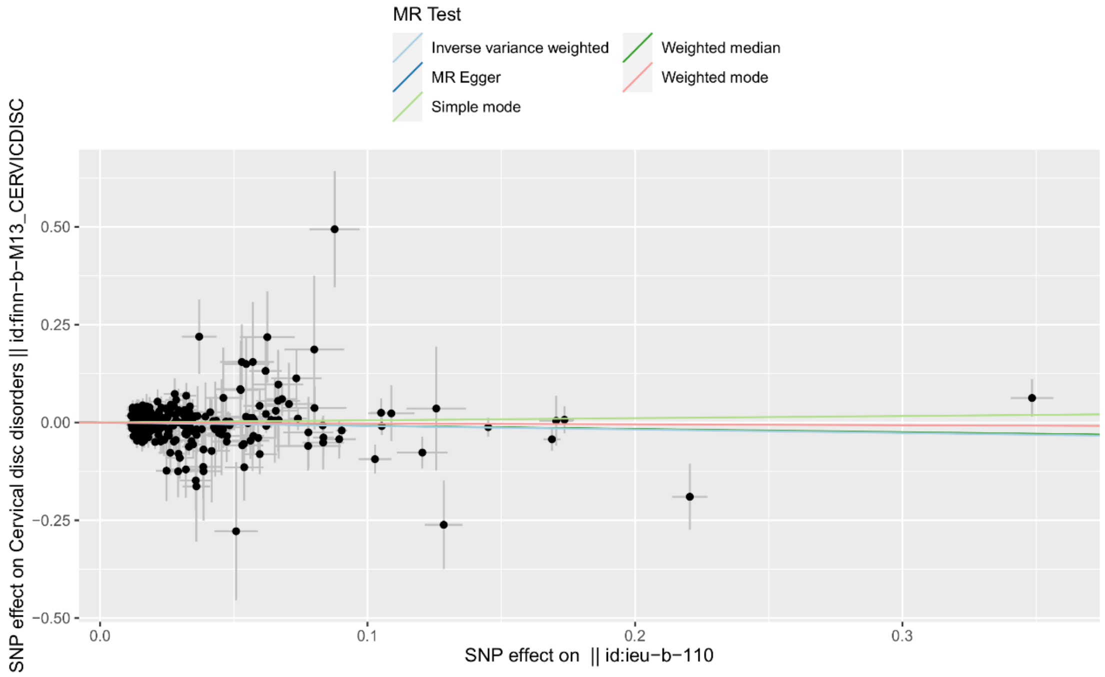


**Supplementary Figure 5.** A scatterplot of variant-LDL-C (x-axis) and variant-cervical spondylosis risk (y-axis) association estimates for LDL-C instruments in univariable MR analysis. The slope of each line corresponds to the effect size obtained by the corresponding MR method.

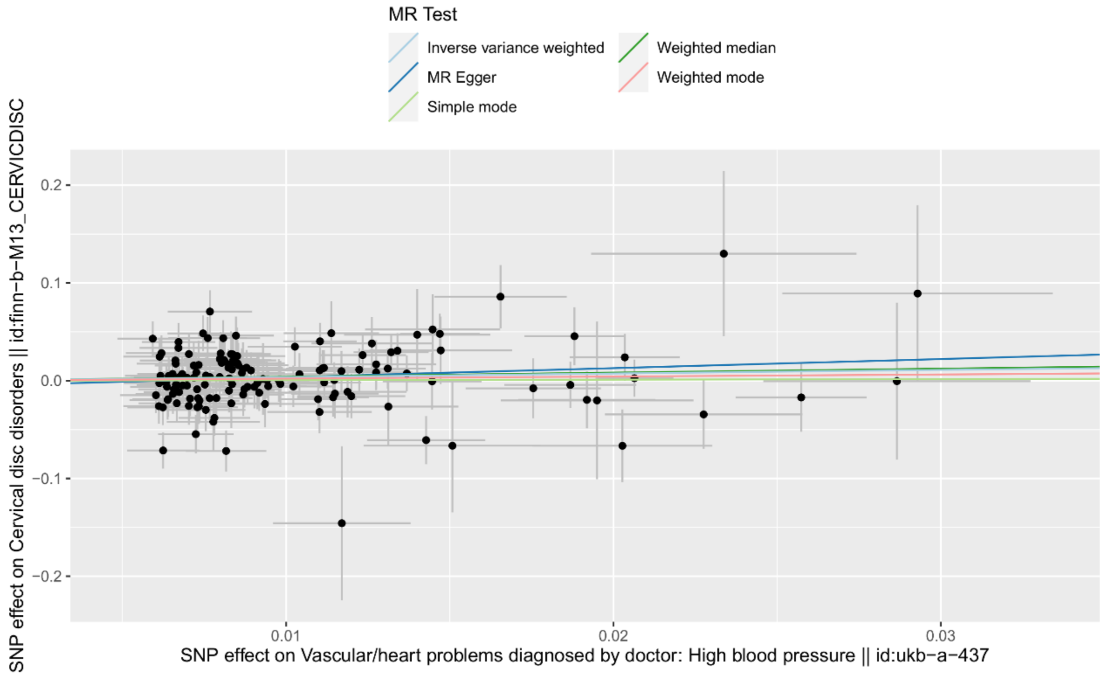


**Supplementary Figure 6.** A scatterplot of variant-BP (x-axis) and variant-cervical spondylosis risk (y-axis) association estimates for BP instruments in univariable MR analysis. The slope of each line corresponds to the effect size obtained by the corresponding MR method.

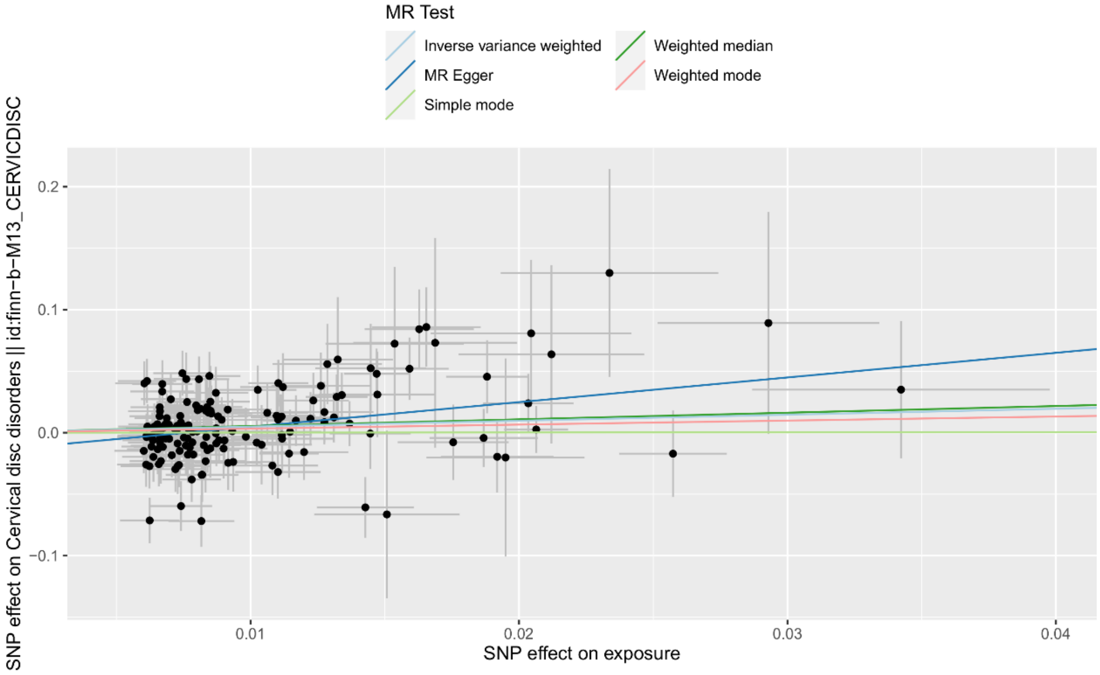


**Supplementary Figure 7.** A scatterplot of variant-smoking (x-axis) and variant-cervical spondylosis risk (y-axis) association estimates for smoking instruments in univariable MR analysis. The slope of each line corresponds to the effect size obtained by the corresponding MR method.

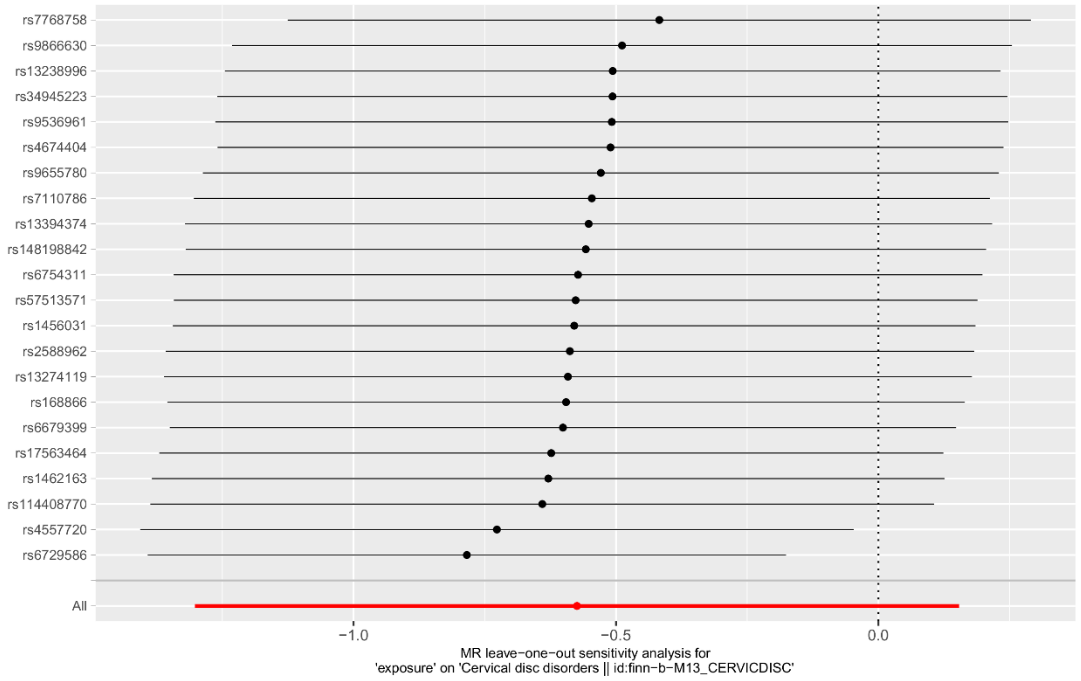


**Supplementary Figure 8.** A "leave-one-out analysis" plot for educational duration instruments in univariable MR analysis.

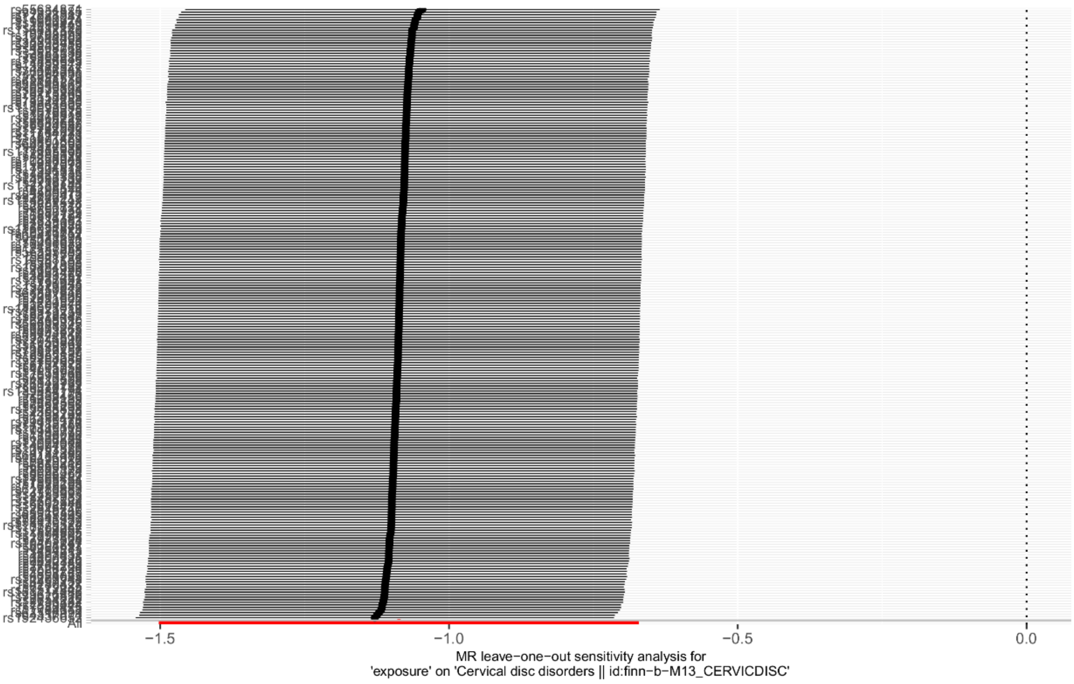


**Supplementary Figure 9.** A "leave-one-out analysis" plot for educational level instruments in univariable MR analysis.

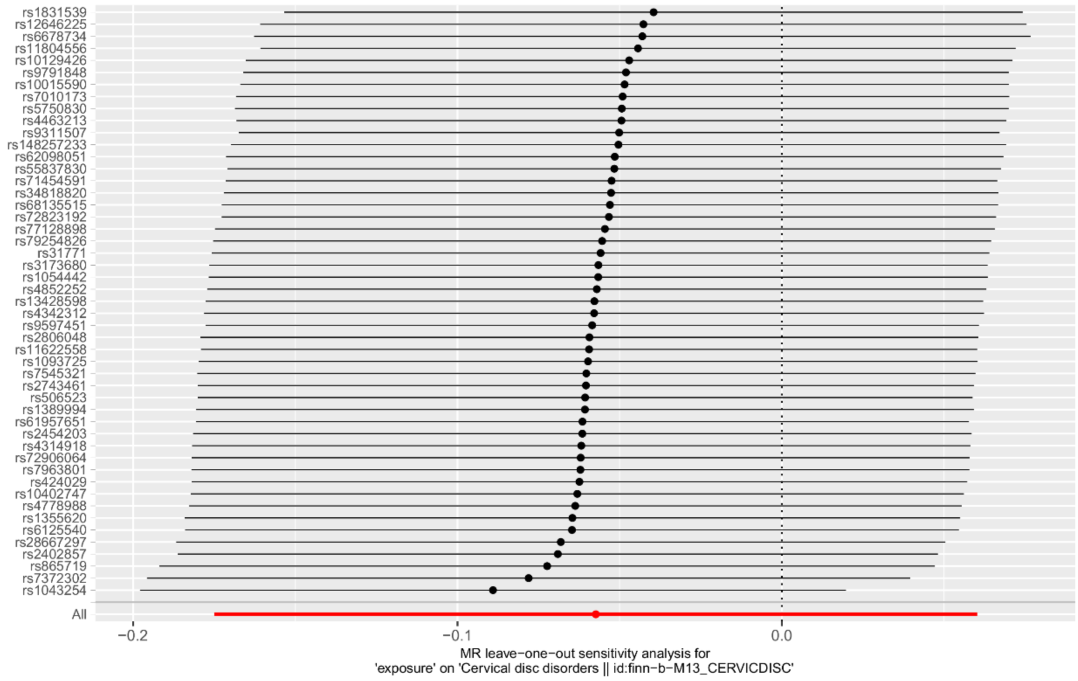


**Supplementary Figure 10.** A “leave-one-out analysis” plot for intelligence instruments in univariable MR analysis

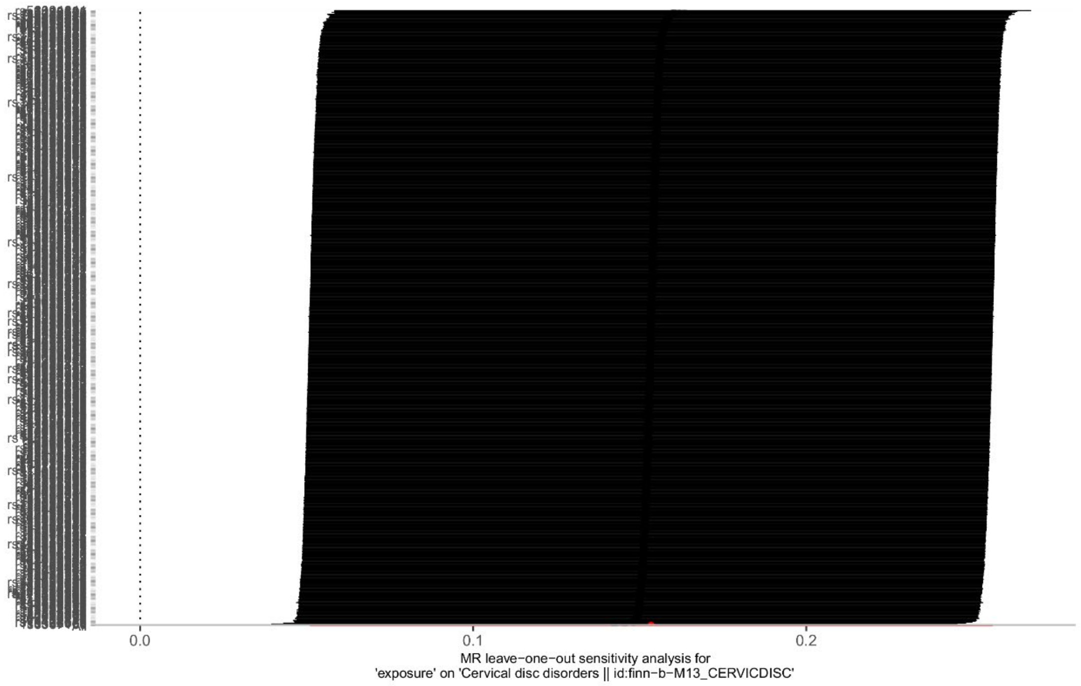


**Supplementary Figure 11.** A “leave-one-out analysis” plot for BMI instruments in univariable MR analysis.

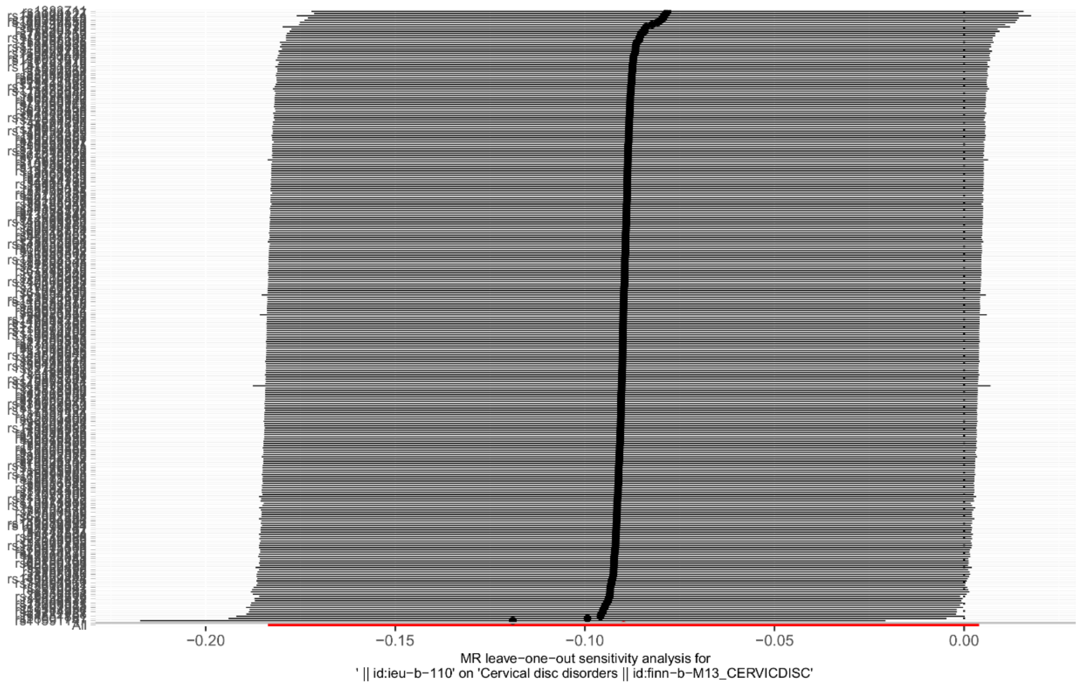


**Supplementary Figure 12.** A “leave-one-out analysis” plot for LDL-C instruments in univariable MR analysis.

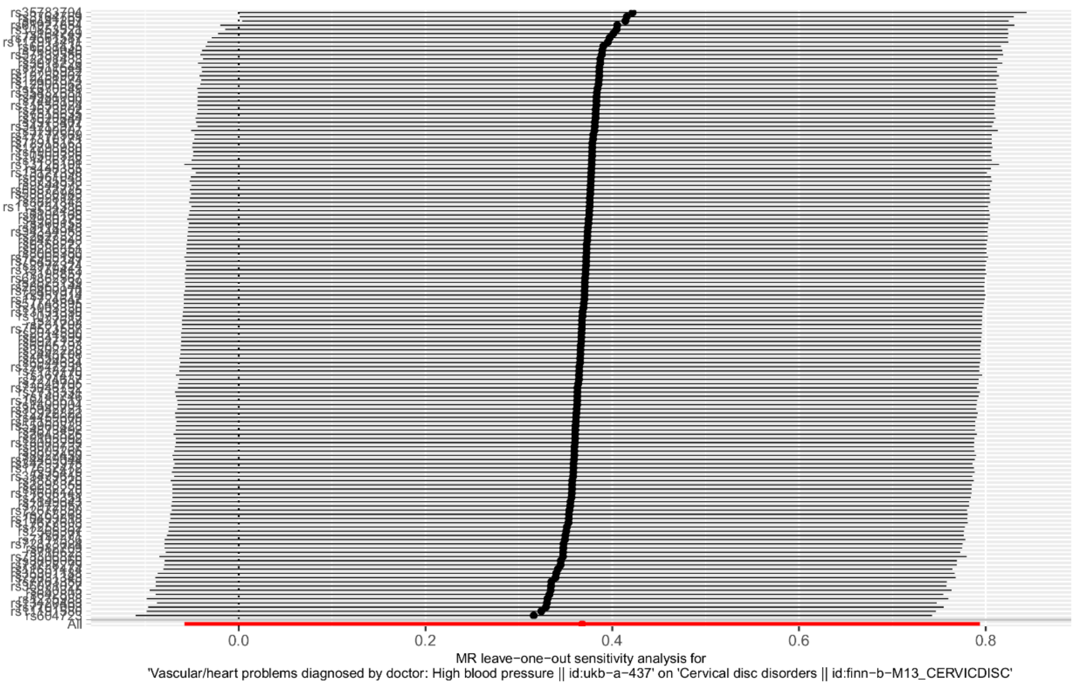


**Supplementary Figure 13.** A "leave-one-out analysis" plot for BP instruments in univariable MR analysis.

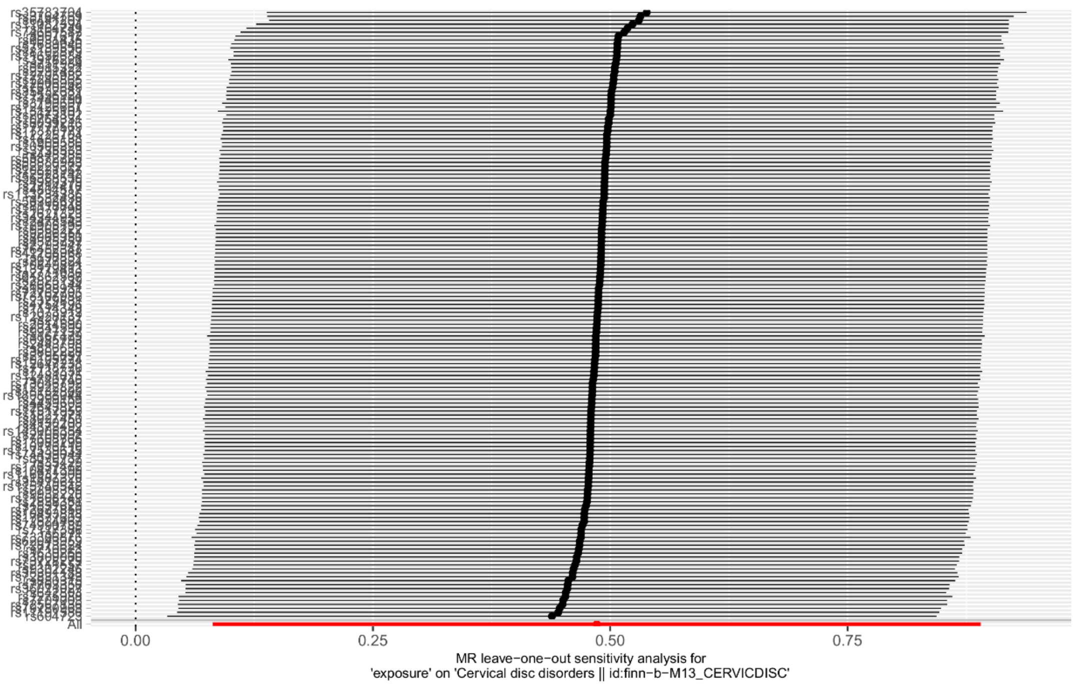


**Supplementary Figure 14.** A "leave-one-out analysis" plot for smoking instruments in univariable MR analysis.
